# Comparative effects of medium‐chain fatty acids or phytobiotics‐based feed additives on performance, caecum microbiota, volatile fatty acid production and intestinal morphology of broilers

**DOI:** 10.1002/vms3.1249

**Published:** 2023-09-02

**Authors:** Necmettin Ceylan, Engin Yenice, İsmail Yavaş, Ali Anıl Çenesiz, Neşe Nuray Toprak, İbrahim Çiftçi

**Affiliations:** ^1^ Department of Animal Science Faculty of Agriculture Ankara University Ankara Türkiye

**Keywords:** broiler, medium‐chain fatty acid, essential oil, alkaloid, caecum

## Abstract

Antibiotic growth promoters have been utilized in broiler nutrition to alleviate the negative effects of the pathogenic microbes to promote performance. However, after the prohibition of antibiotics because of the increasing disclosure related to public health issues, various products have been developed as alternatives. This study was carried out to determine the effects of medium‐chain fatty acids (MCFAs) or phytobiotics (essential oils [EOs] and alkaloids [ALKs]), blended feed additives on the growth performance, jejunum histomorphology, and cecal microbiota of broiler chickens. A total of 765 male Ross 308 chicks were randomly distributed into 5 experimental groups, each having 9 replicates with 17 chicks. The experimental procedures were as follows: a control group without supplementation (T1); control group+ MCFAs and EOs blend (T2); control group+ different EOs blend (T3); control group+ ALK sanguinarine (T4); and control group+ EOs and ALK piperine mixture (T5). The results showed that, broilers fed with MCFAs blended with EOs had significantly greater body weight gain during overall period in comparision to the control and T3 groups. Further, only MCFAs blended with EOs group significantly improved jejnum morphology in comparison with the control group (*p* ≤ 0.05). Besides, the MCFAs blended with EOs group significantly elevated propionate, acetate and butyrate concentration, and decreased the concentration of branch chain fatty acids in caecum (*p* ≤ 0.05). The results indicated that, the combination of MCFAs and EOs seems to have improvement effects and could be preferred as an efficient feed additive in broiler production.

## Graphical abstract

This manuscript aims to investigate different herbal feed additives as alternative to antibiotic growth promoters (AGPs). For this purpose, medium‐chain fatty acids (MCFAs) or phytobiotics (essential oils [EOs] and alkaloids [ALKs]) and blended feed additives have been used. The supplementation of MCFAs combined with EOs improved growth performance, the balance of microbiota in favour of *Lactobacillus* spp. and the surface area of jejunum and SCFA concentration. In brief, MCFAs with EOs have a strong potential as an alternative feed additive for AGP in feeds.

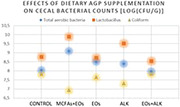

## 1 INTRODUCTION

The gastrointestinal tract (GIT) of poultry is a very critical integrated system that is composed of the functionality of the gastrointestinal barrier, efficient digestion and absorption, active immune status and diet. Besides, the GIT is also colonized by complex microbial communities (Gong et al., 2007). *Escherichia coli, Clostridium, Salmonella, Eimeria* and *Campylobacter* spp. are the dominating pathogens living in the GIT that is composed of significant risks to bird gut health. Antibiotic growth promoters (AGP) have been utilized in broiler nutrition to alleviate the negative effects of the pathogenic microbes to promote the growth of birds due to enhancing effect on GIT microbiota, reducing bacterial fermentation, decreasing the thickness of the GIT wall and suppressing microbial dissimilation and controlling outbreaks of enteric diseases (Broom, 2018). However, after the prohibition of AGP by the EU and the United States, China also put a ban on the use of AGP in 2020 (Wen et al., 2022) because of the increasing disclosure of antibiotic resistance in pathogenic microorganisms related to public health issues (Oliveira et al., 2020).

Removal of AGP in broiler feeds has raised the prevalence of diseases so having a detrimental influence on the yield and health of animals (Aljumaah et al., 2020). Therefore, various products have been developed as alternatives to AGP, such as probiotics, prebiotics, phytobiotics and organic acids (OAs), and their combinations that could be supplemented to the poultry feed to increase growth performance, meliorate intestinal villus integrity, support higher nutrient digestibility or decrease the spread of diseases (Fascina et al., 2012).

The medium‐chain fatty acids (MCFAs) consisting of 6–12 carbon chains are among the most promising as an option to antimicrobial utilization in animals due to their nutritional, physiological and antimicrobial properties (Khatibjoo et al., 2018). MCFAs, namely capric, caproic, caprylic and lauric acids, are more efficiently absorbed and utilized than saturated long‐chain fatty acids and have antimicrobic features, as demonstrated in birds and mammals (Del Alamo et al., 2007; Zentek et al., 2011). MCFAs have strong antibacterial effects due to their capability to pass through bacterial membranes (Dierick et al., 2002). Previous research demonstrated that MCFA has a bactericidal effect by changing the cell permeability, resulting in the loss of the cell membrane's integrity and effectively killing the cell (Kim & Rhee, 2016). Moreover, the antimicrobial potentiality of MCFAs is assumed to surpass short‐chain fatty acids (Hermans et al., 2010). MCFA supplementation also decreased body fat deposition (Wang et al., 2017) and increased breast meat production (Shokrollahi et al., 2014). However, there is a deficiency of consistency on the influences of MCFAs on growth efficiency and gut‐associated criteria. It may be supposed that bird age, microbial load and strain, the amount and sort of MCFAs as well as practical conditions contributed to differences among research results (Çenesiz & Çiftci, 2020).

Phytobiotics or botanicals as other most promising alternatives are composed of natural bioactive components of plant origin, including terpenoids, alkaloids, glycosides and phenolics (Shad et al., 2014). Plant extracts, more commonly known as essential oils (EOs), are composed of lots of complex constituents (Russo et al., 1998) that can be categorized into a set of low molecular weight aliphatic hydrocarbons and a group of terpenoids. Research studies managed in birds have shown the antibacterial effect of EOs against microorganisms, such as *Clostridium perfringens* and *E. coli*, potentially showing a lowered risk of the incidence of necrotic enteritis and colibacillosis (Jamroz et al., 2005; Mitsch et al., 2004). EOs also show antioxidant, anti‐carcinogenic, anti‐inflammatory and hypolipidemic properties (Viuda‐Martos et al., 2010) and may favourably promote gut functions by stimulating endogenous digestive secretions (Jang et al., 2007; Manzanilla et al., 2004). The accumulative effects of EOs on the bioavailability of nutrients and on modulation of the gut microbiota eventually resulted in better performance. There are several research studies showing the positive effects of EOs (Irawan et al., 2020; Peng et al., 2016) on gut health and the performance of chickens; however, the effects are varied and inconsistent (Ipcak & Alcicek, 2018; Shanmugavelu et al., 2004; Tiihonen et al., 2010).

Other phytobiotics extracted from plants are quaternary benzo[*c*]phenanthridine sanguinarine and piperine alkaloids (AKLs) and also have antimicrobial (Eisenberg et al., 1991; Reddy et al., 2004), inflammatory (Firatli et al., 1994; Pradeeb & Kuttan, 2004) and immune‐modulatory (Bussabong et al., 2021) effects. Tschirner (2004) determined that sanguinarine (S‐AKL) and piperine (P‐AKL) supplementations in swine and poultry diets promote growth, increase feed intake (FI) and decrease amino acid degradation. However, subchronic studies on rats supported the negligible evidence of the treatment‐related toxicity of sanguinarine by doses greater than 30 mg/kg/day (Becci et al., 1987), and 120 and 180 mg/kg of piperine supplementation was found to be toxic to liver tissue and decreases the absorption surface of the small intestine, whereas 60 mg/kg of piperine was safe when fed to broilers (Cardoso et al., 2012), which suggest using them at a certain level.

Indeed, studies and above‐mentioned information indicates that MCFAs, EOs and ALKs work differently. Therefore, the above‐mentioned natural compounds either alone or along with each other, such as MCFAs with EOs, EOs with ALKs or EOs with ALKs and OAs, have been gaining interest in poultry production as natural growth promoters for the last decade to obtain their synergic or additive effects on the intestinal health and growth performance. Synergistic, beneficial effects of multiherbal extract components on the inhibition of microbial growth and promotion of productive performance and gut health of broilers were observed (Karukarach et al., 2016). But although various natural additives are developed and applied in the feed industry in different forms and combinations by different commercial companies, the comparison of such natural products in commercial forms on broiler production is seldom been compared, and whether the combinations such as MCFAs with EOs, or a single group of phytobiotics, perform better having synergetic effects needs further examination. Therefore, this study investigated the comparative effect of commercially available in our region and successfully used as natural supplements in practical conditions for the poultry nutrition of MCFAs, EOs and ALKs alone or with each other on growth performance, caecum microbiota, volatile fatty acid production and jejunum morphology of chickens.

## 2 MATERIALS AND METHODS

### 2.1 Birds and housing

A total of 765 1‐day‐old male broiler chicks (Ross 308) were weighed and randomly assigned to 45‐floor pens (1.15 m^2^, 1.20 × 0.95 m^2^ each) containing 17 chicks (14.81 birds/m^2^ stocking density), each equipped with a plastic hanging feeder and nipple drinkers placed on wood shavings. Feed in a mash form and water were provided ad libitum throughout the experiment. Room temperature and lighting were applied according to breeder recommendations (Aviagen, 2018) via automatic heating, cooling and ventilating systems.

### 2.2 Experimental design and diets

The study was executed according to a completely randomized design. One‐day‐old chicks were randomly distributed into 5 treatments with 9 replications having 17 chicks: (1) a control diet (T1); (2) the control diet supplemented with a mixture of MCFAs and EOs (T2) composed of caproic, caprylic and capric acids obtained from palm oil with thymol and trans‐anethole (M‐prove, Nutrition Sciences N.V. Belgium); (3) the control diet supplemented with a blend of EOs (T3) composed of thymol and trans‐anethole (BIOSTRONG 510, Delacon Biotechnik GmbH); (4) the control diet supplemented with an ALK (T4) composed of 1.5% benzophenanthridine alkaloid sanguinarine derived from *Macleaya cordata* (Nusan 1500, Agromed Austria GmbH); and (5) the control diet supplemented with a blend of EOs and ALK (T5) composed of thymol, eugenol, piperine and benzoic acid (CRINA Poultry Plus, DSM Nutritional Products Ltd). Starter (0–10 days), grower (11–24 days) and finisher (25–41 days) diets based on maize and soya bean meals were developed to meet Ross 308 strain suggestions (Aviagen, 2019) (Table 1). In all nutritional phases, the natural additives for T3–T5 treatments were supplemented at 0.15, 0.05 and 0.3 g/kg respectively, whereas T2 was supplemented at 1.2 and 0.7 g/kg in the starter and grower phases, respectively.

**TABLE 1:** Ingredients and composition of control (basal) diets (T1) for different feeding periods of broiler chickens.

Ingredients (g/kg)	Starter (d0–10)	Grower (d11–24)	Finisher (d25–41)
Corn (6.2% CP)	489.86	499.52	515.15
Soya bean meal (47% CP)	378.1	322.4	270.7
Barley (12% CP)	40.0	70.0	90.0
Soya oil	31.7	42.3	52.8
Sunflower meal (33.5% CP)	30.0	40.0	50.0
Limestone	7.47	6.46	5.49
Dicalcium phosphate	7.30	4.34	2.79
dl‐Methionine	3.59	3.15	2.64
Salt	2.68	2.67	2.00
l‐Lysine HCl	2.14	2.01	1.90
Mineral premix^a^	2.00	2.00	1.53
l‐Threonine	1.47	1.24	1.30
Phytase	1.20	1.22	1.20
Sodium bicarbonate	1.03	1.20	1.02
Vitamin premix^b^	1.00	1.00	1.00
Choline chloride (75%)	0.46	0.49	0.48
**Nutrients (%)** ^c^			
Crude protein	23.17 (23.01)	21.23 (21.27)	19.42 (19.13)
Metabolizable energy (kcal/kg)	3000	3095	3190
Ether extract	5.67 (5.90)	6.75 (6.69)	7.82 (7.76)
Crude fibre	3.22 (3.06)	3.61 (3.44)	3.77 (3.53)
Calcium	0.90	0.78	0.66
Available phosphorus	0.45	0.39	0.33
Digestible methionine	0.66	0.60	0.55
Digestible lysine	1.28	1.15	1.03
Digestible methionine + cysteine	0.95	0.87	0.80
Digestible arginine	1.44	1.30	1.18
Digestible threonine	0.86	0.77	0.69
Digestible valine	0.93	0.85	0.77
Digestible tryptophan	0.25	0.23	0.21
Electrolyte balance poultry (mEq/kg)	265.6	245.4	225.0

^a^ Supplied the following per kg of complete feed: 120 mg manganese (Mn oxide); 1.5 mg iodine (Ca iodate); 25 mg iron (Fe sulphate); 16 mg copper (Cu sulphate); 110 mg zinc (Zn oxide); 0.3 mg selenium (Na selenite).

^b^ Supplied the following per kg of complete feed: 4.13 mg retinyl acetate; 125 μg cholecalciferol; 100 mg dl‐α‐tocopheryl acetate; 3.5 mg menadione; 3.5 mg thiamine; 9 mg riboflavin; 20 mg calcium d‐pantothenate; 65 mg niacin; 0.02 mg vitamin B12; 2.2 mg folic acid; 4.5 mg pyridoxine; 0.22 mg biotin.

^c^ Means that calculated values were given outside of parenthesis and analysed values were inside of parenthesis.

All the feedstuffs sampled for analysis were separated at the amount needed for the experiment and put into nylon bags. They are stored under good conditions till the experiment starts. The main feedstuffs, such as corn, barley, soya bean meal and sunflower meal and the experimental diets were analysed for proximate (AOAC, 2005), amino acid and N‐corrected clear metabolizable energy content by near‐infrared reflectance spectroscopy (Evonik Nutrition & Care GmbH). Dietary feed additives of each treatment diet were first mixed for 60 s in a 3 kg capacity laboratory type mixer (type M5R, Gebrüder Lödige Maschinenbau) to make premix by adding amino acids, vitamins–mineral premix, salt, some dicalcium phosphate and the supplement (i.e. MCFA‐, EO‐ or ALK‐based) of the treatment. Then, the dietary feed ingredients of each treatment feed were mixed together with the premix in a 200‐kg capacity vertical mixer for 4 min after the last ingredient was added in the feed mill before the experiment began.

### 2.3 Performance, carcass, abdominal fat and organ measurements

Body weight (BW) and FI were measured on days initial (0), 11, 25 and 41 of the experiment. BW gain (BWG), feed conversion ratio (FCR) and BW‐corrected FCR (WCFCR) were calculated for 0–10, 11–24, 25–41 and 0–41 days. Mortality was recorded daily and was considered in the calculation of FCR. WCFCR was calculated by allowing for a three‐point reduction in FCR for every 100 g BWG increase vs. control:

$$\mathrm{WCFCR}=\mathrm{FCR}-\left(\left(\mathrm{BWGtr}-\mathrm{BWGcontr}\right)/\left(100\right)\right)\times 0.03$$

where BWGtr is the BWG of birds in the treatment group; BWGcontr is the BWG of birds in the control group (Dersjant‐Li et al., 2014). All measurements were made for each replication on a pen base.

At the end of the experiment, two birds per pen close to the mean pen weight were chosen for procedures. Each chicken was weighed and leg‐banded for identification. Each chicken was killed by cutting the jugular vein and defeathered by a rotary picker following bleeding. Abdominal and visceral fats were removed. Then, weights of some visceras (pancreas, liver, proventriculus and gizzard), carcass and carcass parts (thigh, drumstick and breast), intestine (length from gizzard through ceacal junction) and abdominal fat were obtained and calculated as a fraction of individual live BW. Thigh, drumstick and breast as bone in and skin on were weighed and calculated as a fraction of BW.

### 2.4 Intestinal morphological analyses

In order to determine the morphological parameters of each slaughtered bird, 5 cm long parts of the jejunum (middle of the jejunum towards duodenum) were taken and flushed with saline solution. After fixing in 10% neutral buffered formalin for 72 h, jejunal samples were embedded in paraffin wax. Sections with a thickness of 4 μm were taken on a glass slide using a microtome. Images were taken with a computer‐supported imaging system (ImageJ; Laboratory for Optical and Computational Instrumentation) connected to a light microscope (Zeiss Axio Lab.A1 Microscope and Zeiss AxioCam ICc 5 Camera; Cacr Zeiss AG). Villus height (VH) was analysed from the tip of the villus to the junction of the villus and crypt, and the crypt depth (CD) was measured from its base up to the region of transition between the crypt and villus.

Surface area was estimated using the formula = (2*π*) x (VW/2) × (VL) in which VW is the villus width and VL is villus length (Solis de los Santos et al., 2005).

### 2.5 Short‐chain fatty acid determination of cecal content

Short‐chain fatty acid (SCFA) concentration was analysed on day 41 (two birds per replicate). Cecal contents were collected and pooled per replicate pen. Frozen samples from cecal digesta were measured for SCFA concentration by gas chromatography (Zhang et al., 2003).

### 2.6 Cecum‐selected bacterial load determination

Cecal contents of two birds from each pen slaughtered at the end of the experiment on day 42 were collected. The samples of cecal contents were taken in sterile plastic bottles and were immediately placed on ice until the analysis was conducted later on the corresponding day. The cecal microbiota was analysed using a culture technique (Choi et al., 2009). The microbial groups analysed were coliforms (MacConkey agar [105465, Merck]); lactic acid bacteria were enumerated using MRS agar (110660, Merck) and total aerobic counts by using Nutrient agar (105450, Merck).

### 2.7 Statistical analysis

The statistical model was as follows: *y_ij_* = *μ* + *α_i_* + *β_j_* + *£_ij_*, in which *μ* is mean, *α_i_* is the effects of treatments, *β_j_* is effects of block and *£_ij_* is the effect of experimental error. All data originated in the current study were first applied for the normality test. Then, data that appeared normally distributed were analysed by ANOVA using the general linear model procedure of MINITAB 18 software (Minitab Ltd.) in a randomized complete block design, with the cage being defined as a replicate experimental unit. The mortality data were subjected to a chi‐square test. Probability values ≥0.05 were considered significant, and differences among treatments were separated by the Tukey HSD test, whereas 0.05 < *p* < 0.10 was considered a tendency.

## 3 RESULTS

### 3.1 Growth performance, abdominal fat, carcass parameters and organ measurements

The effects of MCFA‐ or phytobiotics‐based blends of the four different feed additives on the performance of birds are presented in Table 2. During the starter and the grower periods, the supplementation of the MCFA‐ and EO‐based blend (T2) resulted in significantly higher BWG in comparison to T1 (control) and T5 (EOs and ALK blend) and compared with T1 and T3 (EOs blend) (*p* ≤ 0.05) during the entire trial period. FCR was significantly improved only during the starter period with the supplementation of MCFAs and EOs blended in comparison to the unsupplemented group (*p* ≤ 0.05). However, any of the supplemented herbal feed additives over the control diet did not significantly influence FI and mortality of broilers (*p* > 0.05) during all of the periods under study.

**TABLE 2:** Effects of dietary supplementation with medium‐chain fatty acid (MCFA)‐, essential oil (EO)‐ and/or alkaloid (ALK)‐based feed additives on growth performance of broilers.

	Treatments						
Performance parameter	T1	T2	T3	T4	T5	Pooled SEM	*p* Value
**Starter** (age 0–10 days)							
Initial BW, g	43.26	43.27	43.28	43.27	43.28	0.08	1.000
BWG, g	221.85^b^	237.22^a^	230.75^ab^	230.74^ab^	225.95^b^	1.68	0.049
FI, g	284.53	295.72	289.00	293.07	288.69	1.80	0.192
FCR, g:g	1.285^a^	1.248^b^	1.253^b^	1.271^ab^	1.278^ab^	0.007	0.047
Mortality, %	0.65	0.00	0.65	0.65	0.00	0.22	0.685
**Grower** (age 11–24 days)							
BWG, g	869.66^b^	901.00^a^	872.02^ab^	889.28^ab^	867.40^b^	7.38	0.048
FI, g	1149.64	1189.14	1160.74	1172.77	1150.79	7.94	0.440
FCR, g:g	1.322	1.321	1.332	1.3207	1.327	0.003	0.622
Mortality, %	0.65	0.65	0.65	0.00	0.65	0.23	0.820
**Finisher** (age 25–41 days)							
BWG, g	1541.6	1576.0	1509.5	1555.8	1563.6	10.43	0.119
FI, g	2544.0	2583.9	2482.5	2550.0	2559.0	17.14	0.400
FCR, g:g	1.650	1.641	1.646	1.640	1.637	0.008	0.981
Mortality, %	1.31	1.96	0.65	0.00	0.00	0.40	0.604
**Overall** (age 1–41 days)							
BWG, g	2640.6^b^	2735.1^a^	2631.9^b^	2687.7^ab^	2667.4^ab^	13.06	0.031
FI, g	3974.7	4103.4	3953.3	4031.3	4003.4	22.91	0.226
FCR, g:g	1.505	1.500	1.502	1.499	1.501	0.003	0.991
WCFCR, g:g	1.507	1.485	1.508	1.493	1.497	0.005	0.382
Mortality, %	2.61	2.61	1.96	0.65	0.65	0.56	0.649

*Note*: T1: control; T2: T1 plus MCFA and EO blend; T3: T1 plus EO blend; T4: T1 plus ALK blend; T5: T1 plus EO and ALK blend. Means within the same column without the same superscript (a, b) are significantly different (*p* ≤ 0.05). Values presented are average of nine replicates.

Abbreviations: BW, body weight, BWG, body weight gain; FI, feed intake; FCR, feed conversion ratio; WCFCR, weight‐corrected feed conversion ratio based on weight gain of control group; SEM, standard error of the mean.

The EOs and ALK blend (T5) supplementation tended to have a lower carcass yield than other treatments except for control group (*p* < 0.10). Additionally, this group of broilers (T5) caused significantly lower breast meat yield than the T1–T3 treatments (*p* ≤ 0.05). Other carcass parameters (thigh and drumstick rates) and abdominal fat deposition were not significantly affected by the treatments (*p* > 0.05) (Table 3). Additionally, the proportional organ weights (liver, pancreas, proventriculus and gizzard) and length of intestine were found to be similar for all treatments (Table 4).

**TABLE 3:** Effects of dietary supplementation with medium‐chain fatty acid (MCFA)‐, essential oil (EO)‐ and/or alkaloid (ALK)‐based feed additives on carcass parameters and abdominal fat (as percentage of live body weight, %) at 42 days of age.

Treatments	Carcass yield	Thigh	Drumstick	Breast	Abdominal fat
T1	76.70^ab^	20.36	10.59	27.43^a^	1.05
T2	76.83^a^	20.20	10.20	27.90^a^	1.04
T3	77.01^a^	20.22	10.54	27.72^a^	1.04
T4	77.17^a^	20.53	10.39	27.33^ab^	1.00
T5	75.75^b^	20.48	10.42	26.29^b^	1.05
Pooled SEM	0.217	0.159	0.051	0.173	0.033
*p* Value	0.071	0.877	0.144	0.030	0.982

*Note*: T1: control; T2: T1 plus MCFA and EO blend; T3: T1 plus EO blend; T4: T1 plus ALK blend; T5: T1 plus EO and ALK blend. Means within the same column without the same superscript (a, b) are significantly different (*p* ≤ 0.05), whereas 0.05 < *p* < 0.10 was considered a tendency. Values presented are average of nine replicates.

Abbreviation: SEM, standard error of the mean.

**TABLE 4:** Effects of dietary supplementation with medium‐chain fatty acid (MCFA)‐, essential oil (EO)‐ and/or alkaloid (ALK)‐based feed additives on digestive organ development (as percentage of live body weight, %) at 42 days of age.

Treatments	Liver	Pancreas	Proventriculus	Gizzard	Length of intestine
T1	2.36	0.231	0.396	1.60	7.13
T2	2.40	0.236	0.406	1.62	6.93
T3	2.38	0.229	0.404	1.58	7.08
T4	2.38	0.234	0.391	1.64	7.15
T5	2.43	0.245	0.411	1.65	7.07
Pooled SEM	0.025	0.003	0.005	0.021	0.090
*p* Value	0.940	0.729	0.811	0.801	0.954

*Note*: T1: control; T2: T1 plus MCFA and EO blend; T3: T1 plus EO blend; T4: T1 plus ALK blend; T5: T1 plus EO and ALK blend.

Abbreviation: SEM, standard error of the mean.

### 3.2 Jejunum morphology

Supplementation with different feed additives significantly influenced the jejunum morphology in broilers (Table 5). Significant improvement was observed only with the MCFA‐ and EO‐blended additive (T2) for VH, villus surface area and VH/CD ratio in compared with to the unsupplemented group (T1) (*p* ≤ 0.05). The MCFA‐ and EO‐blended mixture also caused significantly higher VH than T4 and T5, villus width and surface area than T3 and T5, goblet cell number than T5 and lower crystal depth than T5 (*p* ≤ 0.05). Besides, the addition of ALK (T4) also had significantly higher CD and villus width than T5, whereas EO blend (T3) caused higher villus width and VH/CD than T5 (*p* ≤ 0.05). Additionally, the lowest VH, villus surface area and goblet cell number belonged to the T5 treatment (EO and ALK blend).

**TABLE 5:** Effects of dietary supplementation with medium‐chain fatty acid (MCFA)‐, essential oil (EO)‐ and/or alkaloid (ALK)‐based feed additives on jejunum morphology of broiler chickens at 42 days of age.

Treatments	Villus height (μm)	Crypt depth (μm)	Villus width (μm)	VH/CD	Villus surface area (mm^2^)	Number of goblet cell
T1	1308.7^b^	129.7^ab^	136.1^abc^	10.17^bc^	0.56^bc^	153.5^ab^
T2	1484.1^a^	119.5^b^	152.2^a^	12.47^a^	0.71^a^	173.2^a^
T3	1387.6^ab^	126.0^ab^	129.4^b^	11.39^ab^	0.53^bc^	160.5^a^
T4	1347.4^b^	122.9^b^	142.6^ab^	11.01^ab^	0.61^ab^	155.9^ab^
T5	1263.3^b^	134.8^a^	120.7^c^	9.30^c^	0.48^c^	135.1^b^
Pooled SEM	25.52	1.89	3.47	0.32	0.02	3.83
*p* Value	0.029	0.050	0.050	0.008	0.005	0.047

*Note*: T1: control; T2: T1 plus MCFA and EO blend; T3: T1 plus EO blend; T4: T1 plus ALK blend; T5: T1 plus EO and ALK blend. Means within the same column without the same superscript (a–c) are significantly different (*p* ≤ 0.05). Values presented are average of nine replicates.

Abbreviation: SEM, standard error of the mean; VH/CD, villus height/crypt depth ratio.

### 3.3 Volatile fatty acids and microbiota contents of the caecum

The effects of four dissimilar feed additives on cecal SCFAs are shown in Table 6. The addition of MCFAs blended with EOs (T2) into broiler diets significantly elevated the amounts of cecal acetate, propionate, butyrate and total SCFA production over the control treatment (T1), and acetate, butyrate and total SCFAs in comparison to the other treatments (T3–T5), whereas ALK supplementation (T4) had resulted in an increased production of acetate, propionate and total SCFAs compared with the control group (T1) (*p* ≤ 0.05). The variations between the groups in terms of valerate and isovalerate were detected to be insignificant (*p* > 0.05). Besides, the control group had a tendency to lower isobutyrate level than T2 and T4 groups (*p* < 0.10).

**TABLE 6:** Effects of dietary supplementation with medium‐chain fatty acid (MCFA)‐, essential oil (EO)‐ and/or alkaloid (ALK)‐based feed additives on concentration of ceca short‐chain fatty acids (SCFAs) (μmol/g of dry digesta).

Treatments	Acetate	Propionate	Butyrate	Isobutyrate	Valerate	Isovalerate	BCFA	Total SCFAs
T1	25.50^c^	9.64^b^	6.96^bc^	0.92^b^	1.12	1.03	3.02^b^	45.17^c^
T2	37.95^a^	13.23^a^	9.31^a^	1.15^a^	1.13	0.98	2.27^c^	63.75^a^
T3	25.36^c^	9.75^b^	7.62^bc^	1.06^ab^	1.23	1.03	3.93^a^	46.05^c^
T4	29.08^b^	12.50^a^	7.88^b^	1.08^a^	1.26	1.18	3.77^a^	52.98^b^
T5	25.44^c^	9.94^b^	6.76^c^	1.05^ab^	1.23	1.13	3.83^a^	45.54^c^
Pooled SEM	0.930	0.289	0.229	0.024	0.041	0.040	0.120	1.296
*p* Value	<0.001	<0.001	<0.001	0.080	0.790	0.433	<0.001	<0.001

*Note*: T1: control; T2: T1 plus MCFA and EO blend; T3: T1 plus EO blend; T4: T1 plus ALK blend; T5: T1 plus EO and ALK blend. Means within the same column without the same superscript (a–c) are significantly different (*p* ≤ 0.05), whereas 0.05 < *p* < 0.10 was considered a tendency. Values presented are average of nine replicates.

Abbreviation: BCFA, branched‐chain fatty acids (iso‐butyric12‐me‐butyric1iso‐valeric); SEM, standard error of the mean.

The MCFAs blended with EO addition significantly increased the total aerobic bacteria account and *Lactobacillus* in the caecum of broilers in comparison to the control and T5 groups (*p* ≤ 0.05). However, any other phytobiotics‐based feed additive (T3–T5) had no significant influence on the cecal aerobic bacteria, *Lactobacillus* and coliform bacterial population (Table 7).

**TABLE 7:** Effects of dietary supplementation with medium‐chain fatty acid (MCFA)‐, essential oil (EO)‐ and/or alkaloid (ALK)‐based feed additives on cecal bacterial counts [log (cfu/g)] at 42 days of age.

Treatments	Total aerobic bacteria	*Lactobacillus*	Coliform
T1	8.03^b^	8.78^bc^	7.83
T2	9.10^a^	9.89^a^	6.94
T3	8.50^ab^	8.73^b^	7.65
T4	8.39^ab^	9.52^ab^	7.31
T5	7.97^b^	8.55^c^	7.85
Pooled SEM	0.136	0.149	0.153
*p* Value	0.048	0.019	0.287

*Note*: T1: control; T2: T1 plus MCFA and EO blend; T3: T1 plus EO blend; T4: T1 plus ALK blend; T5: T1 plus EO and ALK blend. Means within column without the same superscript (a–c) are significantly different (*p* ≤ 0.05). Values presented are average of nine replicates.

Abbreviation: SEM, standard error of the mean.

## 4 DISCUSSION

The current study aimed to explore the effects of four different commercial feed additives based on MCFAs and phytobiotics commonly used in broiler nutrition to enhance the growth performance and intestinal health of broilers. Although there are plenty of studies displaying the positive effects of the phytobiotics on the performance of chickens (Diaz‐Sanchez et al., 2015; Kikusato et al., 2021; Weber et al., 2012; Zeng et al., 2015; Zhu et al., 2019), the addition of the three additives composed of thymol and trans‐anethole blend (T3), ALK sanguinarine (T4) and a blend of EOs (thymol and eugenol) enriched with ALK piperine (T5) had no significant effect on all of the performance parameters studied in current study, which are in agreement with some other experiments reporting that the application of different types, concentrations or combinations of EOs, such as thymol, eugenol and trans‐anethole, and ALKs such as sanguinarine and piperine did not affect the BW, BWG, FI and FCR results, and sometimes even makes it worse (Abudabos & Alyemni, 2013; Barreto et al., 2008; Onimisi et al., 2017). The response of the phytobiotics in birds to improve growth performance and feed efficiency is inconsistent, which has been imputed to the dose, source and type of EO or ALK, dietary and management conditions that have always not been examined systematically (Chowdhury et al., 2018; Su et al., 2018). Some oils may be irritating to the mucous lining of the intestine, resulting in inflammation. Thus, it is critical to appropriately select, compose and amount of the phytobiotics supplementation (Su et al., 2018).

However, the application of MCFAs combined with EOs (thymol and trans‐anethole) (T2) significantly increased the overall BWG by 3.6% and 3.8% compared with the control and the mixture of EOs (T3), respectively, whereas FCR was better than the control chicks only during the starter period. Synergistic effects of MCFAs combined with EOs on broiler production in terms of improved final BWG and FCR have been documented (Bansal et al., 2018; Nguyen et al., 2018; Timmerman et al., 2006), which may be in agreement with this study even though there was no difference in FI among the treatments and in comparison to the control. Conversely, some research studies showed no performance improvement when broilers received MCFAs in comparison to the control broilers and broiler‐fed antibiotics (Nichols et al., 2021; Shokrollahi et al., 2014).

Carcass and breast meat ratio seems to be reduced by the inclusion of the blend of EOs and ALK piperine (T5). Although the other treatments had no significant influence on the carcass parameters, this might be related to the benzoic acid present in the blend of EOs and ALK piperine. Jozefiak et al. (2008) hypothesized that more than 0.1% benzoic acid supplementation could be the reason for the reduced growth performance of broilers after 2 weeks of age because of its metabolic pathway conjugated with ornithine. Benzoic acid supplementation may cause an arginine deficiency, leading to disturbed protein deposition in breast meat because dietary arginine is the source of ornithine in the fowl. It should be emphasized that almost all of the plasma ornithine in the poultry is derived from arginine metabolism. In the Isabel and Santos (2009) study, breast weight was significantly greater in chickens fed supplements of clove and cinnamon oils than in other experimental groups. They suggested that although dietary EOs occur to have a beneficial effect on breast yield, the interactions between OA salts and EOs remain unclear. Contrary to our result, Shokrollahi et al. (2014) reported that supplementation with MCFA treatments had a higher breast weight in comparison to unsupplemented group. The researchers estimated that MCFAs stimulate the secretion of insulin, and then, glucose intake by breast muscle increases, and the resulting energy from the oxidation of glucose can be used for protein synthesis.

The improvement and structural integrity of intestinal mucosa reflecting gut health in the poultry production is of considerable importance in achieving ideal growth rates and FCR (Xue et al., 2020). In this study, the improved growth performance in final BWG and FCR because of the inclusion of MCFAs combined with EOs might be attributed to the synergism between the MCFAs and the EOs to modulate the intestinal structure and ecosystem of broilers, thus leading to an increased absorption of nutrients. This argument was mainly derived and supported by the results of this study obtained from intestinal measurements, indicating an improved epithelial structure of the jejunum and the favourable production of SCFAs by cecal microbiota. Villi and crypts are significant constituents of the small intestine, and their geometry provides an indicator of the absorptive capacity of the intestine (Heydarian et al., 2020). Longer villus and shorter crypt are usually equated with excellent gut health, high absorptive efficiency and healthier intestinal tract of chickens. Chiang et al. (2010) suggested that a greater VH/CD ratio indicates the proper digestion and absorption of nutrients. The results of this study showed that VH, villus surface area, VH/CD ratio and a number of goblet cells in the jejunum were highest with the application of the MCFAs are in agreement with the previous studies (Abudabos et al., 2017; Rodríguez‐Lecompte et al., 2012; Sultan et al., 2015), which might increase the nutrient absorption and disease resistivity and then account for the promoted BWG and FCR in the MCFA treatment. The improved growth performance may also be attributed to the well‐known mode of action for EOs via the stimulation of endogenous digestive secretions (e.g. enzymes mainly in the pancreas) and maintaining intestinal epithelial structures (Manzanilla et al., 2004), thus leading to increased digestibility of nutrients as recently confirmed by Su et al. (2018) with the application of a thymol, carvacrol and cinnamaldehyde mixture. Higher activity and production of the pancreatic and digestive enzymes were exhibited in birds that received thymol (Jang et al., 2007) or oregano EOs, whereas Masouri et al. (2017) also reported an increase in digestive juices, which has antimicrobial effects by supplementing EOs.

The clearly increased morphological structure of the jejenum with the addition of MCFA and EO combination, as the authors measured, may be attributed to the synergetic action of MCFAs and EOs related to their strong antimicrobial activities on pathogens, and the nutritional contribution of MCFAs to epithelial cells of the intestine. Dai et al. (2021) reported that MCFAs not only have an antibacterial effect but also provide energy for the gut epithelial cells. Monoglyceride form of MCFAs can reach the hindgut by passing through the stomach of chickens and be decomposed into butyric acid under the action of microbes, thus improving intestinal morphology and promoting intestinal immunity (Guo et al., 2008). Oxygen radicals liberated during digestive processes act on the superficial mucous of the intestine and shorten the intestinal villi. The EOs may protect the villi from oxidative damage by stimulating the activity of antioxidant enzymes such as superoxide dismutase (Chowdhury et al., 2018), which may further contribute to the growth of the intestine. Yet alone EOs, ALK or EOs with ALK groups have no effects on the morphological structure of broilers. Zhang et al. (2022) mentioned that although the oral solutions of EO‐ or *M. cordata*–based additives alone have limited effects on intestinal morphology on broilers, their combination increased the efficiency on intestinal morphology. In literature it is stated that the efficiency of plant‐based additives may be changed according to such factors: a content of bioactive ingredients, processing methods, physicochemical properties, plant parts, using dose and form and interactions with other feed ingredients (Jang et al., 2007; Zhang et al., 2022). On the other hand, the improved structure of jejunum can also be associated with the elevated production of SCFAs in the caecum of broilers due to the inclusion of MCFAs based, and the ALK sangunarine additives because of the movement of butyrate from the caecum to the ileum and jejunum through antiperistalsis and then promoting small intestinal development (Liao et al., 2020).

The microbiota in the caecum express high metabolic activity in the GIT of chickens and ferment indigestible carbohydrates in feed components to produce a series of metabolites. Among the various metabolites, SCFAs, mainly acetate, propionate and butyrate, have received extensive attention because of their positive effects on health (Liu et al., 2017). These SCFAs are key issues in healthy gut and are required for intestinal functionality and the integrity of the intestine (Meimandipour et al., 2010). Supplementation of MCFAs and EO thymol and trans‐anethole combination in the current experiment resulted in higher acetate, propionate butyrate production thus leading to the highest total SCFAs, and lower branched‐chain fatty acid (BCFA) concentrations in the caecum of broilers than the unsupplemented control treatment. The SCFA production by gut microbiota, particularly butyric acid, may benefit the host bird by supplying energy, accelerating gut epithelial cell proliferation, epithelial barrier integrity and intestinal immunity as well as collagenous and non‐collagenous protein syntheses in mucosa (Ma et al., 2012). Moreover, SCFAs are also proposed to improve gut barrier function, although the underlying mechanism of beneficial effects of butyrate on the function of tight junction is obscure. Myosin light‐chain kinase might play a crucial role in the regulation of tight junctions as indicated by Liu et al. (2017). However, excessively accumulated isovaleric and isobutyric acids indicate an unusual course of digestion and fermentation. Extremely high amount of non‐absorbed proteins or amino acids reaching the caecum may lead to the increased production of these putrid acids (Śliżewska, 2020). The BCFAs are released from the fermentation of proteins in the hind gut, particularly sloughed intestinal cells (Cardona et al., 2005). In this experiment, the reduction in caecal BCFA production by the inclusion of MCFAs and EOs blend in the feed might be due to a decrease in bacterial fermentation of branched‐chain amino acids because of better protein utilization reflected with the improved growth performance in the current trial. Although there is no data available on the effects of MCFAs on SCFA production in the ceca of broilers, Tiihonen et al. (2010) showed the beneficial effect of the tyrol (15 g/t) and cinnamaldehyde (5 g/t) combination had their major effects on caecal metabolites resulting in an increase in mainly butyrate concentration at 20 and 41 days of age of broilers, which is in accordance with this study. Besides, the increased concentration of acetate, propionate and butyrate and reduced BCFA concentration in the caecum may be further associated with the antimicrobial properties of MCFA–EO blend inclusion in this study by limiting the growth of pathogens, but promoting the beneficial bacterial colonization in the gut.

Certain Eos, such as thymol, carvacrol and eugenol, can permeabilize and depolarize the cytoplasmic membrane of the bacteria (Lippens et al., 2005). This means that the cell membrane is weaker, and that the MCFAs can enter much easier in the cell. In this way, it can be speculated that the synergistic effects between the MCFAs and the EOs by modifying the gut microflora in favour of beneficial bacteria such as *Lactobacillus* spp. could result in the increased concentrations of acetate, propionate butyrate. *Lactobacillus, Butyricimonas* and *Roseburia* genera are known to produce SCFAs and have shown beneficial effects on the host's development and health (Farnworth, 2005). Liao et al. (2020) demonstrated that *Salmonella* was inversely related to acetic acid, butyric acid and isovaleric acid, meaning that these SCFAs might inhibit the growth and invasion of *Salmonella* in the caecum. In the same study, a positive correlation between *Lactobacillus* and acetic acid was shown in the ileum, meaning intestinal health could be improved by *Lactobacillus* through producing some SCFAs. In this study, MCFAs blended with thymol and trans‐anethole increased the growth of *Lactobacillus* and aerobic bacteria in comparison with the control treatment in the caecum of broilers. This result is in accordance with that reported by Meimandipour et al. (2010) who suggested that lactate, produced by *Lactobacillus* spp. in the cecal digesta, promotes the growth of butyrate‐producing bacteria, which notably increases the cecal butyrate concentration. Other herbal additives used in this study had limited effects on bacterial microbiota in the caecum even the worst in EO‐ and ALK‐derived groups. Using herbal additives in poultry nutrition should be according to specific animal age, contamination status and health status of chicks (Granstad et al., 2020) because the effectiveness of these additives relies on these factors and many other factors stated before, when using should be kept in mind for animals.

## 5 CONCLUSION

The results of this study showed that the MCFA combined with EOs significantly improved the growth performance of the broiler chickens in comparison to the unsupplemented control and other phytogenic commercial counterparts, which was supported not only by improved the balance of gut microbiota in favour of *Lactobacillus* spp., but also by the improved surface area of jejunum and SCFAs concentration. Thus, we can conclude that MCFA combined with EOs have potential as an alternative feed additive for AGP in broiler chickens.

## AUTHOR CONTRIBUTIONS

All authors are either employed by or associated with a government agency or university, the primary function of which is research and education.

## CONFLICT OF INTEREST STATEMENT

The authors declare that there are no conflicts of interest.

## FUNDING INFORMATION

This research received no specific grant from any funding agency in the public, commercial or not‐for‐profit sectors.

## ETHICS STATEMENT

The authors confirm that the ethical policies of the journal, as noted on the journal's author guidelines page, have been adhered to and the appropriate ethical review committee approval has been received. The US National Research Council's guidelines for the Care and Use of Laboratory Animals were followed. The Animal Ethics Committee of the Ankara University (Approval number: 2021‐94) approved all procedures involving animals.

### PEER REVIEW

The peer review history for this article is available at https://publons.com/publon/10.1002/vms3.1249.

## Data Availability

No.
